# Impacts of chronic jetlag on compensatory angiogenesis in 5XFAD mice

**DOI:** 10.1002/alz70855_100465

**Published:** 2025-12-23

**Authors:** Cathryn A Cutia, Mary E Harrington

**Affiliations:** ^1^ Smith College, Northampton, MA, USA

## Abstract

**Background:**

Cerebrovascular insufficiency and circadian disruption occur in many Alzheimer's disease (AD) patients. These comorbidities appear in preclinical phases of AD and persist over the progression of the disease. How cerebrovascular insufficiency and circadian disruption interact and contribute to AD remains poorly understood. Here, we seek to test the hypothesis that circadian disruption during an early period of amyloid beta deposition disrupts the cerebrovasculature, thus interfering with endogenous angiogenic response to hypoperfusion which sustains cerebrovascular insufficiency and exacerbates cognitive function in AD.

**Method:**

3‐month‐old 5xFAD animals (*n* =  20) and control animals (*n* =  20) were exposed to either 3 months of jet lag (6‐hour phase advances every week) or 12:12 light:dark cycles. At 6 months of age, each mouse underwent a bilateral carotid artery stenosis surgery to induce cerebral hypoperfusion (*n* =  10) or underwent a sham surgery (*n* =  10). Following a month of hypoperfusion, the mice were euthanized, and brain tissue was evaluated for angiogenesis via CD31 stain or for white matter reduction via Luxol blue stain

**Result:**

Following 3 months of jet lag, 5xFAD animals show higher rhythmicity (*p* = 0.007) and amplitude (*p* = 0.003) of locomotor activity when compared to their beginning values. These mice did not show changes in their circadian period (*p* = 0.43). Control animals show no change in mean rhythmicity (*p* = 0.3) or amplitude (*p* = 0.4) over time. Due to the ongoing nature of data collection, angiogenesis measures have yet to be evaluated in these mice, but will be presented.

**Conclusion:**

When exposed to circadian disruption across the highest period of amyloid beta deposition, 5xFAD animals display increased measures of rhythmicity that we propose may be due to a higher response to light. This will be tested in further experiments. This indicates that repeated phase shift exposure during the period of amyloid beta deposition may improve measures of locomotor activity. We will present data on the implications on vascular health and recovery.

